# Indoor Localization System Based on RSSI-APIT Algorithm

**DOI:** 10.3390/s23249620

**Published:** 2023-12-05

**Authors:** Xiaoyan Shen, Boyang Xu, Hongming Shen

**Affiliations:** 1 School of Information Science and Technology, Nantong University, Nantong 226019, China; xiaoyansho@ntu.edu.cn (X.S.); 2110320058@stmail.ntu.edu.cn (B.X.); 2 Nantong Research Institute for Advanced Communication Technologies, Nantong University, Nantong 226019, China

**Keywords:** indoor localization system, received signal strength indication, RSSI-APIT algorithm, ANN

## Abstract

An indoor localization system based on the RSSI-APIT algorithm is designed in this study. Integrated RSSI (received signal strength indication) and non-ranging APIT (approximate perfect point-in-triangulation test) localization methods are fused with machine learning in order to improve the accuracy of the indoor localization system. The system focuses on the improvement of preprocessing and localization algorithms. The primary objective of the system is to enhance the preprocessing of the acquired RSSI data and optimize the localization algorithm in order to enhance the precision of the coordinates in the indoor localization system. In order to mitigate the issue of significant fluctuations in RSSI, a technique including the integration of Gaussian filtering and an artificial neural network (ANN) is employed. This approach aims to preprocess the acquired RSSI data, thus reducing the impact of multipath effects. In order to address the issue of low localization accuracy encountered by the conventional APIT localization algorithm during wide-area localization, the RSSI ranging function is incorporated into the APIT localization algorithm. This addition serves to further narrow down the localization area. Consequently, the resulting localization algorithm is referred to as the RSSI-APIT positioning algorithm. Experimental results have demonstrated the successful reduction of inherent localization errors within the system by employing the RSSI-APIT positioning algorithm. The present study aims to investigate the impact of the localization scene and the number of anchors on the RSSI-APIT localization algorithm, with the objective of enhancing the performance of the indoor localization system. The conducted experiments demonstrated that the enhanced system exhibits several advantages. Firstly, it successfully decreased the frequency of anchor calls, resulting in a reduction in the overall operating cost of the system. Additionally, it effectively enhanced the accuracy and stability of the system’s localization capabilities. In a complex environment of 100 m^2^ in size, compared with the traditional trilateral localization method and the APIT localization algorithm, the RSSI-APIT localization algorithm reduced the localization error by about 2.9 m and 1.8 m, respectively, and the overall error was controlled within 1.55 m.

## 1. Introduction

The utilization of wireless sensor networks (WSN) in indoor localization has gained significance due to advancements in this technology. Compared to GPS localization, the indoor localization system is more localized, and it can function ordinarily in situations where satellite signals cannot be received due to signal blockage [1]. Indoor localization technology plays a crucial role in the security industry, including larceny prevention and mine personnel rescue. In addition to the security field, indoor localization technology is increasingly used in everyday life. For instance, in crowded public places, such as offices and shopping malls, indoor localization can help analyze the movement of people in each scene in order to allocate scene resources more effectively and reduce the cost of energy consumption [2,3,4].

However, indoor localization technology continues to encounter numerous obstacles. When there are more obstacles in the indoor scene between the sensor nodes, the communication path will encounter obstructions, resulting in a sharp decrease in signal strength [5]. At the same time, obstacles cause the wireless signal to be reflected and refracted numerous times in indoor space, and the various components of the electromagnetic wave arrive at the receiving end at different times, resulting in interference and distortion of the original signal, which to some extent also affects the quality of communication between wireless sensors. The evolution of hardware technology does not permit indoor localization systems to circumvent these issues. Consequently, the majority of current research on indoor localization systems focuses on enhancing the precision of localization algorithms.

There have been many studies related to indoor localization algorithms. In [6], indoor localization techniques are described in detail by Faheem Zafari et al. Indoor localization algorithms are mainly classified into two main categories: ranging-based localization algorithms and non-ranging-based localization algorithms [7]. In the study of ranging-based localization algorithms, IM Khan et al. used acceleration sensors in smartphones in combination with RSSI to improve the accuracy of indoor localization algorithms [8]. AOA-based techniques have also been developed in recent years. In [9], A Florio et al. introduced a technique to optimize AOA positioning using phase interferometry. SA Shaikh et al. [10] combined lens antennas with large-scale antenna arrays and proposed a method to optimize AOA localization. In the study of non-ranging-based localization algorithms, H Lu et al. [11] optimized indoor fingerprint localization by using a weighted K nearest neighbors (WKNN) and extreme gradient enhancement (XGBoost) algorithm, resulting in a significant reduction of the localization error.

All of these schemes improve the accuracy of indoor localization, but they have some limitations. Ranging-based localization algorithms require high hardware clock synchronization, and this form of localization algorithm is affected by the environment: if there is more interference in the environment, ranging accuracy will be impacted, leading to a reduction in localization accuracy. Unlike the localization algorithm based on ranging, the localization algorithm not based on ranging does not need to measure the distance information between each anchor and the tag to be tested. This type of algorithm is less affected by the surrounding environment, and its stability is high. However, the workload of collecting information in the early stage is substantial, and because it is based on a locally established database, the adaptability of the algorithm is poor, and it is difficult to apply to scenarios with significant environmental changes, such as the need to move an anchor [12].

In this paper, therefore, we combine ranging and non-ranging localization algorithms with machine learning to enhance the accuracy of indoor localization systems by compensating for the limitations of ranging or non-ranging algorithms alone.

## 2. Indoor Localization System Design

### 2.1. Indoor Localization System Architecture

**Figure 1:**
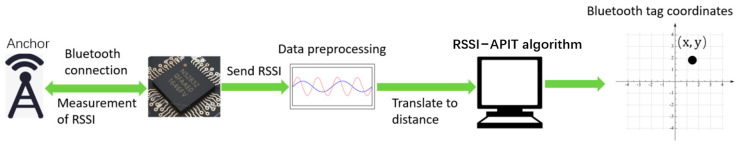
Architecture of the indoor localization system.

Figure 1 illustrates the construction of the indoor localization system as a whole. The anchor is linked to the Bluetooth chip and initiates the measurement of RSSI. Subsequently, the RSSI data obtained at such distance undergo preprocessing. The preprocessing procedure encompasses several steps, including the use of Gaussian filtering, the elimination of outliers through the use of residuals, and the estimation of distance values employing an ANN. The RSSI data that have undergone preprocessing are transformed into distance values, which are subsequently used in conjunction with the RSSI-APIT algorithm to approximate the coordinates of the Bluetooth tag.

### 2.2. Data Preprocessing

A fluctuation of RSSI values is observed in the localization space due to the multipath effect of the signal. This effect causes the RSSI values to vary to a certain extent, even when the anchor and the tag are maintained at a constant distance. In general, the RSSI value at a constant distance conforms to a Gaussian distribution [13,14]. This paper employed Gaussian filtering as a preprocessing technique for the data. Equations (1)–(3) depict the probability density function of *n* RSSI values that have been gathered at the same distance:

(1) $$\begin{array}{c} F\left(RSSI\right)=\frac{1}{\sigma \sqrt{2\pi }}e^{\frac{{\left(RSSI-\mu \right)}^{2}}{2\sigma^{2}}} \end{array}$$

(2) $$\mu =\frac{1}{n}\sum_{i=1}^{n}{RSSI}_{i}$$

(3) $$\sigma =\sqrt{\frac{\sum_{i=1}^{n}{\left({RSSI}_{i}-\mu \right)}^{2}}{n-1}}$$

where μ is the mean and σ is the standard deviation. The Gaussian distribution has a symmetrical probability distribution with a peak at the mean, μ, and the likelihood of distribution decreases as the distance from the mean increases [15]. The function F(RSSI) represents the likelihood that the individual RSSI values are distributed symmetrically around the mean, and a larger probability indicates more reliability of the data.

The application of Gaussian filtering reduces the magnitude of fluctuations in the gathered data. However, it is important to note that certain RSSI values may still exhibit noticeable deviations from the expected or typical values. To preserve the integrity of data collection, it is necessary to eliminate outliers in this particular segment. Residual plots, when used in conjunction with linear regression, are commonly employed to identify outliers [16]. In order to conduct linear regression, it is necessary to first identify and exclude singular RSSI values that significantly differ from the normal range. Let the *i*th RSSI value be ${RSSI}_{i}$ and the *i*th RSSI value, predicted by the linear regression equation, be ${\hat{RSSI}}_{i}$; then, the residual $e_{i}$ is:

(4) $$\begin{array}{c} e_{i}={RSSI}_{i}-{\hat{RSSI}}_{i} \end{array}$$

If the residuals exhibit considerable magnitude, it indicates that the observed data have significantly deviated from the expected value, suggesting the presence of an outlier that should be identified and then eliminated.

To enhance the precision of data preprocessing, this study integrated an ANN into the preprocessing procedure. There exists a multitude of ANNs, encompassing many forms, such as the feedforward neural network (FNN) and recursive neural network (RNN), among others. This paper employed the back propagation (BP) neural network. The BP neural network employs the rapid descent method, using back propagation to iteratively update the weights and thresholds inside the network; hence, minimizing the network error [17]. The BP neural network is comprised of three layers: an input layer, a hidden layer, and an output layer. The artificial neural network (ANN) used in this study comprised an input layer, a hidden layer with 10 neurons, and an output layer. The RSSI value was used as an input, while the matching distance value was used as an output for training the ANN.

### 2.3. RSSI-APIT Localization Algorithm

#### 2.3.1. RSSI Ranging

RSSI ranging is a more commonly used ranging method in WSN. Due to the interference in the environment, a certain degree of attenuation occurs in the propagation of wireless signals, and the attenuation increases with the distance. A mathematical correlation exists between the attenuation and distance, allowing the use of signal attenuation to infer a distance between the location of signal transmission and the point of reception. The Log-Normal signal attenuation model is commonly employed for RSSI ranging in WSN, when the network consists of *N* anchors. This model is typically represented by Equation (5) in the literature:

(5) $$\begin{array}{c} {PL\left(d\right)}_{i}=PL\left(d_{0}\right)-10nlg\left(\frac{d_{i}}{d_{0}}\right)-N_{0} \end{array}$$

The variable $d_{i}$ represents the distance between the *i*th anchor and the Bluetooth tag, with the unit of measurement being meters. The variable ${PL\left(d\right)}_{i}$ represents the signal strength in decibels (dBm) between the *i*th anchor and the Bluetooth tag. The value of $PL\left(d_{0}\right)$ is the reference signal strength, often defined as the signal strength at a distance of 1 m between the anchor and the Bluetooth tag. The environmental attenuation factor, denoted as *n*, represents the impact of the current environment on the signal strength. It is often determined by experimental data analysis, and its value is subject to variation across different experimental circumstances. $N_{0}$ is the Gaussian random noise variable. The linear relationship between $PL\left(d\right)$ and the logarithm of the distance value lgd may be observed from Equation (5). Hence, the $PL\left(d\right)-lgd$ curve can be obtained through the use of linear regression, and the environmental attenuation factor, *n*, can be ascertained based on the slope and intercept of this curve [18].

#### 2.3.2. APIT Localization Algorithm

The APIT localization algorithm is a non-ranging-based approach, distinguishing itself from trilateral localization and angular localization methods. Unlike these methods, APIT does not require the collection of distance or angle information. Instead, it derives the location information of the tags by considering the connectivity between multiple tags and the number of hops between them. This approach effectively mitigates interference from the surrounding environment.

The localization algorithm of the APIT is comprised of two primary phases. In the first step, every three of the N anchors around the tag to be tested are formed into a triangle, so that a total of $C_{N}^{3}$ triangles can be formed. The area of the $C_{N}^{3}$ triangles is evaluated by the use of the intra-triangle point test method. In the event that the tag exhibits a certain direction of movement, if the tag concurrently approaches or recedes from the three vertices of the triangle, it can be inferred that the tag is positioned outside the triangle and, consequently, the triangle fails to meet the required criteria. On the contrary, the object is situated within the confines of the triangle, and the triangle itself adheres to the established standards or requirements. Ultimately, a total of K triangles that adhere to the specified criteria are documented.

**Figure 2:**
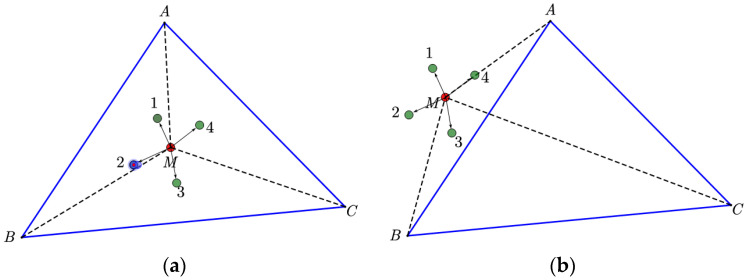
Inside point test method in WSN with triangle. (**a**) Inside point test method in WSN with Bluetooth tag inside the triangle. (**b**) Inside point test method in WSN with Bluetooth tag outside the triangle.

Within WSN, the intercommunication between nodes can be effectively employed to replicate the motion of a tag in a specific trajectory. As depicted in Figure 2a, the tag under examination, denoted as M, establishes communication with node 1 and acquires knowledge that its movement toward node 1 results in distancing from anchor C while approaching anchors A and B. Similarly, it trades information with the rest of the nodes 2, 3, and 4, which ultimately leads to the conclusion that it is placed in a triangle. According to the diagram presented in Figure 2b, when the tag undergoes a movement toward node 1, it simultaneously distances itself from anchors A, B, and C. Conversely, when the tag moves toward node 3, it concurrently approaches anchors A, B, and C. Consequently, it can be concluded that the tag does not reside within the triangle, as stated in [19].

**Figure 3:**
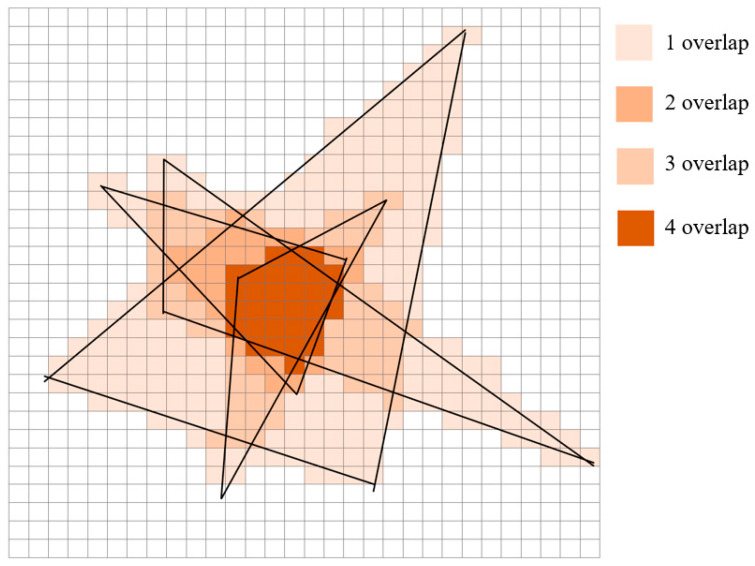
Determining the area of overlap.

The subsequent stage of the APIT technique involves the calculation of the overlapping zone among the eligible K triangles. The resulting center of gravity inside this overlap region is then used as the estimated coordinates for the Bluetooth tag. The determination of the overlapping region can be achieved by using a software-based methodology. The localization region is partitioned into many discrete squares, with each square initially assigned a value of zero. Each triangular shape encompasses a distinct portion of the smaller square, resulting in an increment of 1 in the value attributed to the covered area of the small square. Once the K triangles have been encompassed, the graph of the little square exhibiting the maximum value corresponds to the region of overlap among the K triangles. The accuracy and time cost of localization are influenced by the division of small squares. When the division of small squares is smaller, the accuracy of calculating the overlapping region increases, but the time required also increases. The visual representation depicted in Figure 3 illustrates the assessment of intersecting areas. The area denoted by the numerical value of 4 in the diagram corresponds to the zone of overlap. According to the findings presented in Figure 3, it is evident that the accuracy of the overlapping region is influenced by the dimensions of the small squares. This effect is most pronounced at the periphery of the overlapping zone. When the small squares are set to larger sizes, the edge of the overlapping region becomes increasingly blurred [20].

Suppose the overlapping region *X* can be divided into *n* triangles $X_{1},X_{2},X_{3},\ldots ,X_{n},$ the three vertices of the *i*th triangle are $A_{i}\left(x_{1i},y_{1i}\right),B_{i}\left(x_{2i},y_{2i}\right),\;\mathrm{and}\;C_{i}(x_{3i},y_{3i})$, and the center of gravity ${(G}_{ix},G_{iy})$ of the *i*th triangle is:

(6) $$\begin{array}{c} \left\{\begin{array}{c} G_{ix}=\frac{x_{1i}+x_{2i}+x_{3i}}{3},i=1,2,3,\ldots ,n\\ G_{iy}=\frac{y_{1i}+y_{2i}+y_{3i}}{3},i=1,2,3,\ldots ,n \end{array}\right. \end{array}$$

To determine the area $A_{i}$ using the vector product method, we built a coordinate system using the point $C_{i}(x_{3i},y_{3i})$ as the origin. Subsequently, the area $S_{i}$ is:

(7) $$\begin{array}{c} S_{i}=\frac{\left|\vec{C_{i}B_{i}}\;\times \;\vec{C_{i}A_{i}}\right|}{2} \end{array}$$

where $\vec{C_{i}B_{i}}$ is the vector from $C_{i}$ to $B_{i}$ and $\vec{C_{i}A_{i}}$ is the vector from $C_{i}$ to $A_{i}$. Then, the coordinates of the center of gravity, $O(Z_{x},Z_{y})$, of the overlapping region are:

(8) $$\begin{array}{c} \left\{\begin{array}{c} Z_{x}=\frac{\sum_{n}^{i=1}G_{ix}S_{i}}{\sum_{n}^{i=1}S_{i}}\\ Z_{y}=\frac{\sum_{n}^{i=1}G_{iy}S_{i}}{\sum_{n}^{i=1}S_{i}} \end{array}\right. \end{array}$$

The final estimation coordinates for the Bluetooth tag are determined by considering the center coordinates of the overlapping region.

#### 2.3.3. RSSI-APIT Localization Algorithm

The accuracy of the APIT localization algorithm is significantly compromised when the localization area is extensive, meaning that the accuracy of the algorithm diminishes as the area of the composed triangles increases. This is due to the fact that the final outcome of the APIT algorithm is obtained by estimating the coordinates of the centers of gravity of multiple triangles. Moreover, the localization accuracy is deemed unsatisfactory in instances where the long and short sides of the triangle established by the anchor exhibit proximity to one another. In order to tackle the issue of reduced accuracy in certain scenarios, this study presents an enhanced APIT localization algorithm, called the RSSI-APIT localization algorithm. Building upon the existing APIT localization algorithm, the proposed approach incorporates RSSI-based ranging functionality to narrow down the estimated location area of the tag.

**Figure 4:**
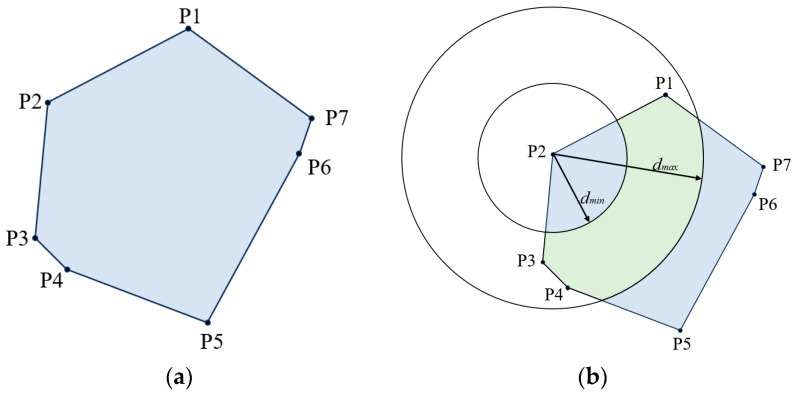
Improvement of the APIT algorithm using RSSI. (**a**) APIT algorithm to obtain overlapping regions. (**b**) Narrowing the localization region using RSSI.

Figure 4a illustrates the region of overlap in Figure 3. The vertices of the overlapping region are denoted as P1, P2, P3, P4, P5, P6, and P7. P2 is the physically existing anchor with the function of communicating with the tag. The remaining vertices refer to the intersection points that are produced when the triangles cross each other. These points are virtual nodes that are obtained through calculation and do not possess the capability to communicate with the tags. The tags in the overlapping area communicate with the anchor at point P2 to derive the current set of RSSI values. The maximum RSSI value, ${RSSI}_{max}$, and the minimum RSSI value, ${RSSI}_{min}$, were extracted from the RSSI values derived after Gaussian filtering and converted into the distance values $d_{max}$ and $d_{min}$ using Equation (5). Using P2 as the circle’s center, $d_{max}$ and $d_{min}$ as its radius, and keeping the two circles in the overlapping region of the arc segment, we can obtain the overlapping region of a section of the circle and use green to represent it, as shown in Figure 4b.

**Figure 5:**
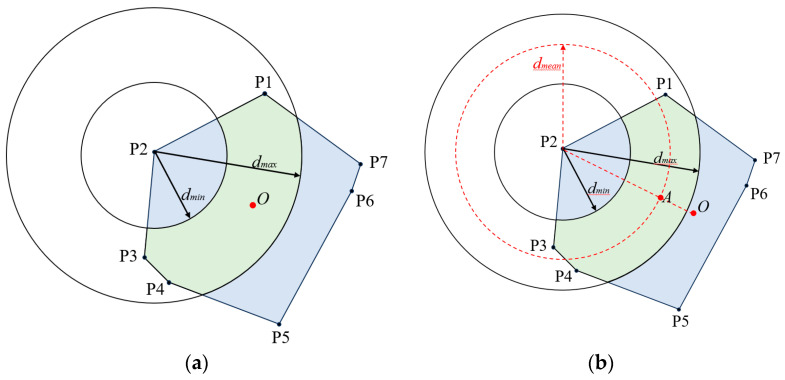
Estimation of localization coordinates using the center of gravity. (**a**) Localization estimation of the Bluetooth tag with the center of gravity inside the circle. (**b**) Localization estimation of the Bluetooth tag with the center of gravity outside the circle.

The APIT localization algorithm is used to determine the center of gravity point O inside the overlapping region. Subsequently, the distance $d_{OP2}$ between point O and P2 is computed. If the condition $d_{min}\leq d_{OP2}\leq d_{max}$ is satisfied, indicating that the center of gravity point O falls within the circle, it can be inferred that the confidence level of the center of gravity is higher. Consequently, the estimated position of the tag is determined to be the center of gravity point O. If the point O is outside the circle, confidence in the center of gravity is low. At this juncture, the average of the current set of RSSI values is computed and converted to the distance value $d_{mean}$. Then, a circle is drawn with P2 as its center and $d_{mean}$ as its radius, retaining the arc in the overlapping region and use red to represent it. The connection between point O and point P2 is established, and the resulting intersection point A is identified as the estimated coordinates of the Bluetooth tag, where it intersects with the arc of the circle. Both cases are illustrated in Figure 5a,b.

**Figure 6:**
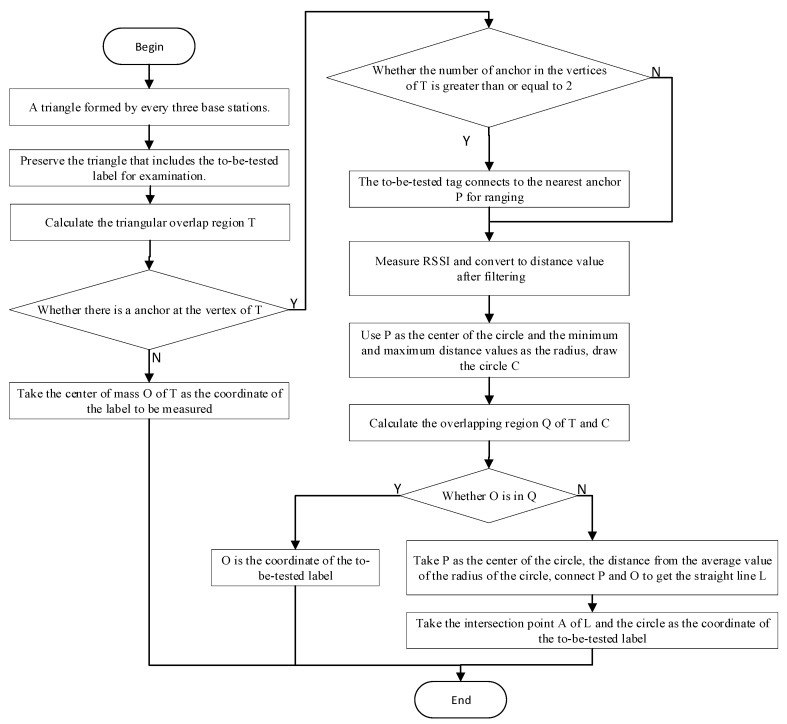
RSSI-APIT localization algorithm flowchart.

If the vertices of the overlapping region do not include communicative anchors, it indicates that the anchors are situated at a considerable distance from the tags, resulting in a diminished level of reliability in the localization information provided by the RSSI values. In this case, the final estimated coordinates of the tag are determined by the APIT localization algorithm, which calculates the center of gravity of the overlap zone. In cases when the overlapping region encompasses multiple communicable anchors, the anchor with the highest RSSI value, indicating closer proximity, is selected as the central point for defining the circular localization area. This localization area is then refined based on the average RSSI values. Figure 6 depicts the flowchart of the RSSI-APIT localization algorithm.

## 3. System Testing

### 3.1. Signal Preprocessing Tests

**Figure 7:**
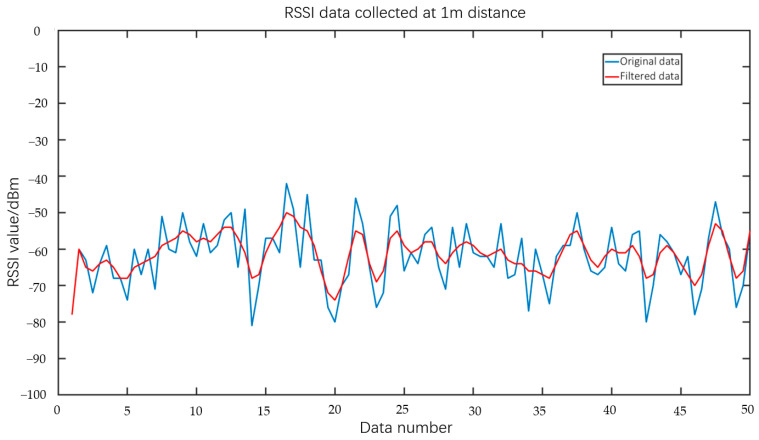
RSSI values for Gaussian filtering.

This study investigated the performance of RSSI ranging experiments conducted within an indoor environment. Initially, the RSSI values were gathered at various distances, ranging from 1 m to 10 m. The RSSI values were collected in increments of 0.5 m, resulting in a total of 50 data points per group. Each group of data was subjected to the application of the Gaussian filter, resulting in the acquisition of the filtered data. Subsequently, the mean value of the filtered dataset was computed to obtain the RSSI value corresponding to the given distance. Figure 7 depicts the curve representing the RSSI value obtained subsequent to the use of Gaussian filtering. The graphic illustrates that the application of Gaussian filtering resulted in a notable reduction in data volatility when compared to the original dataset. This phenomenon resulted in increased stability of the RSSI values obtained at identical distances, hence enhancing the accuracy of distance estimation by the use of average values.

**Figure 8:**
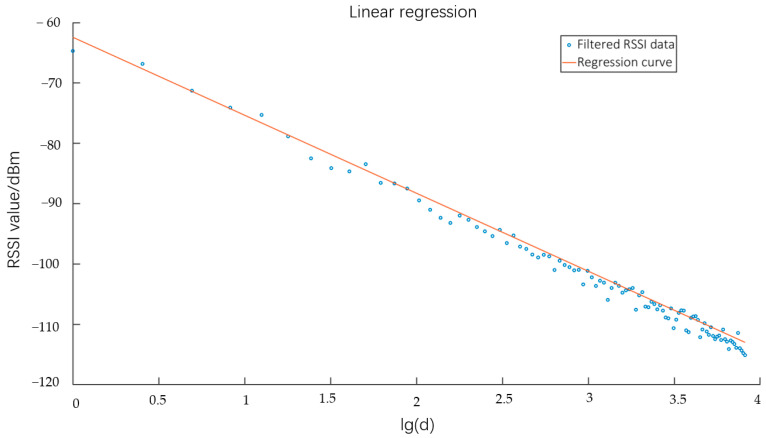
Linear regression plot of RSSI vs. lg(d).

Next, we proceeded with the computation of the residuals, which are defined as the differences between the observed data points and the corresponding predicted values. It is important to note that data points exhibiting excessively large residuals can be classified as singular data. After excluding the singular data, the remaining *n* data points were assigned the variables ${rssi}_{n}$ and $d_{n}$ for the RSSI value and distance, respectively. According to Equation (5), a linear correlation exists between *rssi* and *lgd*. Consequently, linear regression can be employed to analyze the data points $\left(lgd_{1},{rssi}_{1}\right),\left(lgd_{2},{rssi}_{2}\right),\ldots ,(lgd_{n},{rssi}_{n})$. Figure 8 displays the outcomes of the linear regression analysis.

The linear regression figure reveals that, upon preprocessing, the data exhibited a strong match. Following the elimination of singular data, the RSSI data and distance data that remain were preserved and employed for training the ANN. From the linear regression plot, it can be seen that the distribution of RSSI values became more and more dense with the increase of distance, and this phenomenon may lead the neurons in the ANN to enter into the saturation state. To mitigate this behavior, it is imperative to normalize the RSSI and distance measurements before training the ANN [21]. The input ${RSSI}^{*}$ of the ANN is defined as:

(9) $$\begin{array}{c} {RSSI}^{*}=\frac{RSSI-{RSSI}_{min}}{{RSSI}_{max}-{RSSI}_{min}} \end{array}$$

**Figure 9:**
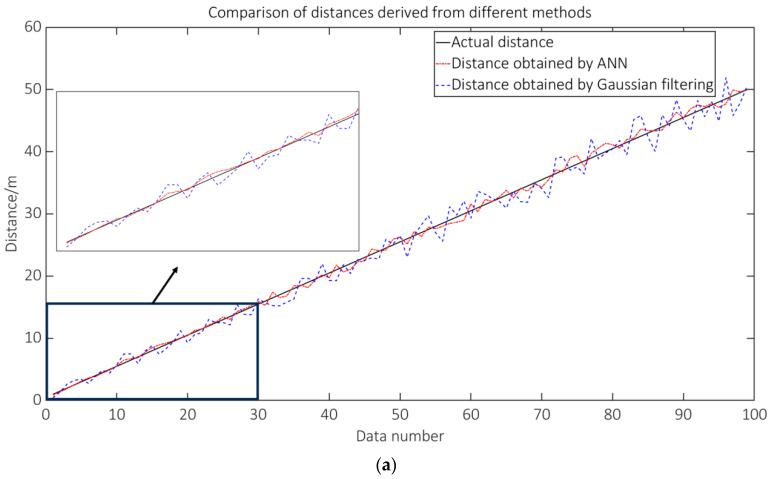

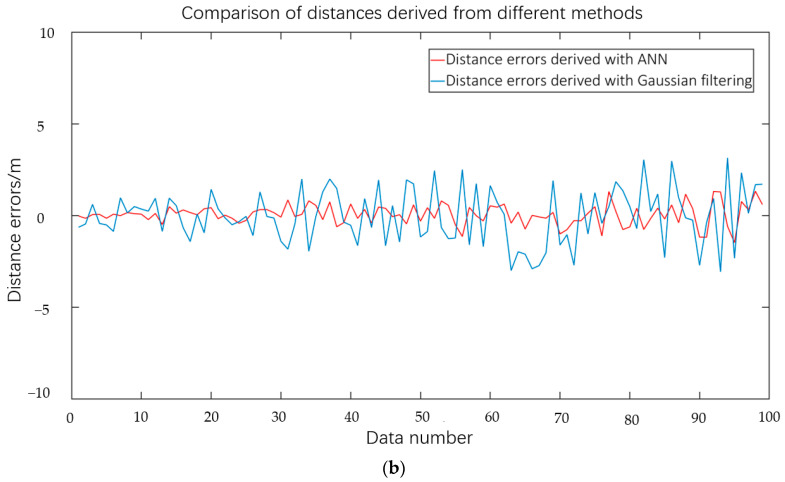
ANN and Gaussian filter ranging. (**a**) Comparison of ANN and Gaussian filter ranging. (**b**) Comparison of ANN and Gaussian filter ranging error.

The variable ${RSSI}^{*}$ represents the normalized RSSI value, whereas ${RSSI}_{max}$ and ${RSSI}_{min}$ denote the maximum and minimum values of the RSSI data, respectively. The distance value was also normalized, and the normalized distance, $d^{*}$, is:

(10) $$\begin{array}{c} d^{*}=\frac{lgd}{lgd_{max}} \end{array}$$

where $d_{max}$ represents the highest value inside the dataset of distance values. The ${RSSI}^{*}$ was used as the input parameter, while the $d^{*}$ was employed as the output parameter for training the ANN. Figure 9a presents the comparison between the distance data obtained from the ANN and the actual distance data, and the comparison between the distance error of the ANN and the Gaussian-filtered distance error is shown in Figure 9b. The distance error was calculated as the discrepancy between the estimated distance and the actual distance and was further categorized into positive and negative values. The graphic demonstrates that the distance values derived through the use of ANN exhibited reduced fluctuations in comparison to the actual distance values. As the distance between data points increased, the errors of both approaches exhibited an increase. However, it is noteworthy that the ANN method consistently demonstrated a lesser error compared to Gaussian filtering across all distances. ANN possess a notable advantage over Gaussian filtering when it comes to longer distances.

### 3.2. RSSI-APIT Localization Algorithm Testing

The localization algorithm was evaluated using MATLAB for simulation purposes, using a configuration of 20 anchors positioned inside a 100 m^2^ region. The entire process was categorized into offline and online phases. The offline phase was responsible for training the ANN. The anchors were arranged before conducting the experiment. The tags to be tested were then randomly distributed inside the localization region for a total of 50 trials. Each anchor measures the RSSI value between itself and the tags to be tested. The RSSI value underwent a process of filtering and averaging before being used as the input for the ANN. Conversely, the actual distance value served as the desired output for the ANN. The network performance was trained using many sets of input and output data.

Once the network training concluded, it transitioned into the online phase. During this phase, each anchor measures the RSSI value between itself and the Bluetooth tag. This measurement occurs at a sampling rate of 20 times per second. The average RSSI value, obtained after filtering, was then used as input for the ANN. The output of the network is the predicted distance value. The determined coordinates of the tag to be measured were estimated using the distance value in conjunction with the localization technique. The phenomenon of localization error in the conducted experiment refers to the measure of the distance between the estimated coordinates and the real coordinates $\left(X_{i},Y_{i}\right)$. It is important to note that the localization error can have both positive and negative values.

(11) $$\begin{array}{c} RMSE=\pm \sqrt{{\left(x_{i}-X_{i}\right)}^{2}+{\left(y_{i}-Y_{i}\right)}^{2}} \end{array}$$

The average localization error is defined as:

(12) $$\begin{array}{c} AVG_{RMSE}=\frac{\sum_{i=1}^{n}{|RMSE}_{i}|}{n} \end{array}$$

where *n* is the number of measured localization error data points.

#### 3.2.1. Experiments with the RSSI-APIT Localization Algorithm

**Figure 10:**
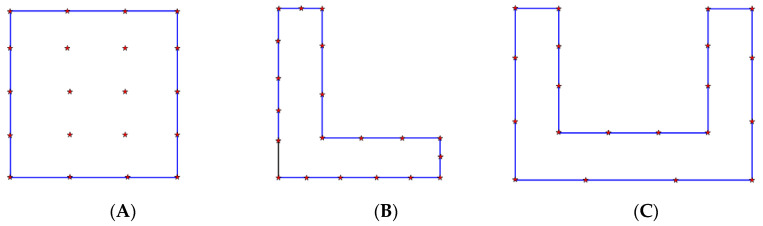
Anchor layout scheme. (**A**) Square area. (**B**) “L” corridor. (**C**) “C” corridor.

To examine the effiscacy of the localization algorithm acryijioss several settings, the experiment encompassed three distinct scenarios. Figure 10 illustrates the spatial distribution of the anchors, denoted by the red pentagrams.

**Figure 11:**
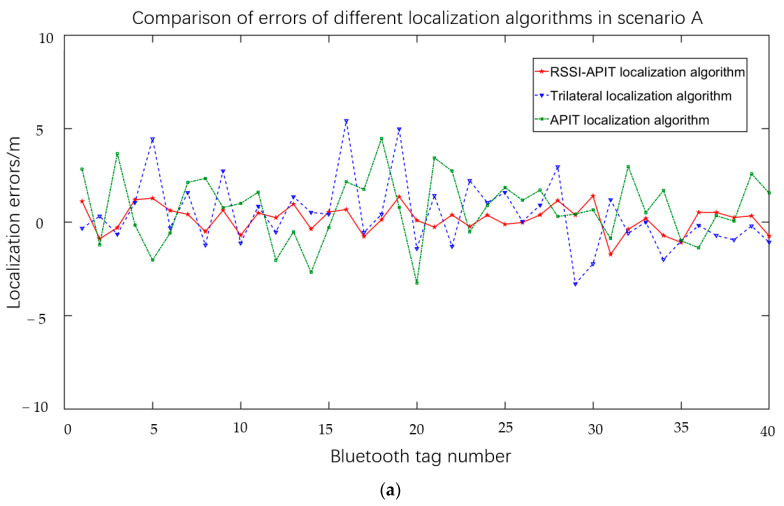

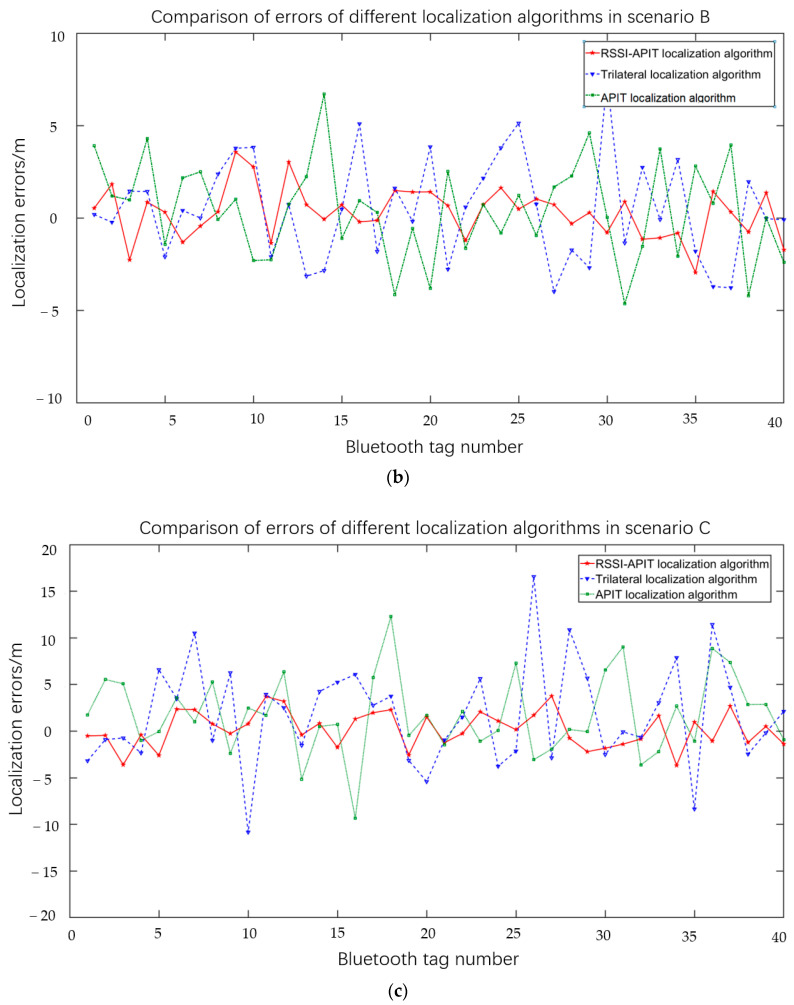

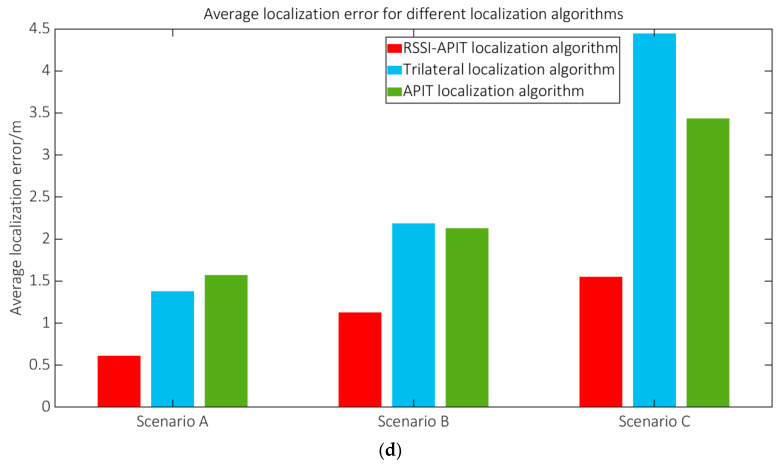
Comparison of different localization algorithms in three scenarios. (**a**) Comparison of errors of different localization algorithm errors in scenario A. (**b**) Comparison of errors of different localization algorithms in scenario B. (**c**) Comparison of the errors of different localization algorithms in scenario C. (**d**) Comparison of the average localization errors of the three localization algorithms in different scenes.

Scenario A represents an arrangement of 20 anchors positioned within a square area. Scenario B, on the other hand, refers to the arrangement of anchors within the “L” corridor, while scenario C pertains to the arrangement of anchors within the “C” corridor. Then, the Bluetooth tag was placed in the localization area 40 times to measure the localization error. Figure 11a illustrates the localization error of several algorithms in scenario A. The figure illustrates that the RSSI-APIT localization algorithm exhibited a more consistent error distribution. Specifically, it was observed that 85% of the evaluated tags demonstrated a localization error within a range of 1.5 m. The error of the trilateral localization algorithm and the APIT localization algorithm exhibited more fluctuations when the localization anchors were uniformly distributed.

The Bluetooth tags were thereafter positioned within scenarios B and C, wherein the efficacy of various localization methods was assessed within these two contexts. The test findings in the two circumstances are depicted in Figure 11b,c, respectively. Scenarios B and C exhibited a greater number of corners and walls, resulting in an increased presence of multipath phenomena in the signal propagation. This effect was particularly pronounced in the more intricate scenario C. The errors exhibited by each of the three localization algorithms experienced varying degrees of amplification, resulting in an overall increase in the volatility of the localization process. The trilateral localization algorithm exhibited the highest level of volatility within the set of algorithms considered. This is primarily due to its heavy reliance on the accuracy of the RSSI measurements. The presence of multipath effects significantly influenced the RSSI value, leading to a substantial decrease in the accuracy of the trilateral localization algorithm. The RSSI-APIT localization algorithm demonstrated efficacy in mitigating data volatility through the use of ANN training. Figure 11d demonstrates that the RSSI-APIT localization algorithm exhibited a smaller average localization error compared to the other two algorithms across various localization scenarios. Notably, in the more intricate scenario C, in comparison to the conventional trilateral localization algorithm and the APIT localization algorithm, the RSSI-APIT localization algorithm demonstrated a reduction in localization error of around 2.9 m and 1.8 m, respectively. Furthermore, the overall error was effectively constrained to within 1.55 m.

#### 3.2.2. Impact of the Number of Anchors on Localization Accuracy

**Figure 12:**
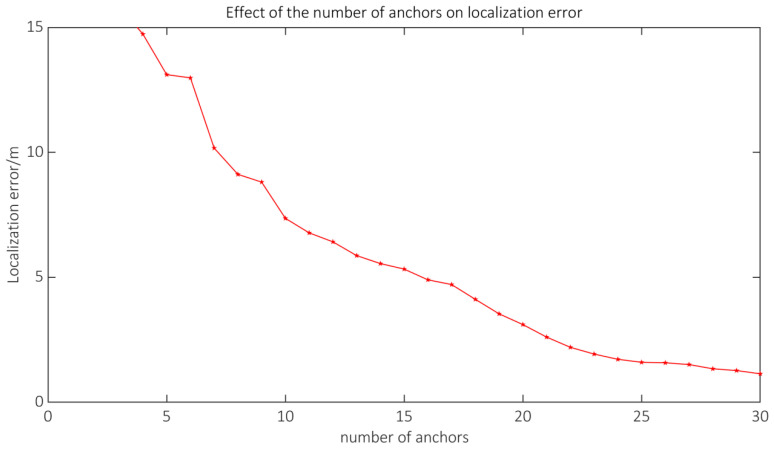
Effect of the number of anchors on the localization error.

The localization accuracy is influenced by the number of anchors in real applications. This section aims to examine the correlation between the quantity of anchors and the precision of localization. In scenario A, a uniform arrangement of 3–30 anchors was considered, followed by a random arrangement of the Bluetooth tags. Each number of anchors was measured 30 times, and the average error value was calculated as the final result. The experimental results are depicted in Figure 12.

The precision of localization was partially influenced by the quantity of anchors. The augmentation in the quantity of anchors diminished the spatial extent of the region wherein the Bluetooth tags were situated, hence enhancing the precision of the ultimate estimation. When the quantity of anchors is limited, enhancing the number of anchors can lead to a notable enhancement in localization accuracy. When the quantity of anchors reached around 15, it was possible to maintain the localization error within a range of 5 m. When the quantity of anchors reached approximately 20, it was possible to decrease the localization error to approximately 2 m. Once the quantity of anchors reached 25, the entirety of the localization area was essentially encompassed, resulting in a marginal enhancement from further increasing the number of anchors. Given the financial implications associated with hardware expenses, it would be more prudent to allocate around 20 anchors in scenario A.

## 4. Conclusions

In this paper, an indoor localization system based on an improved APIT algorithm was studied. The system combines RSSI filtering processing and an artificial neural network, which effectively reduces the volatility of RSSI data. The RSSI-APIT localization algorithm was proposed on the basis of the APIT localization algorithm, and the simulation results showed that compared with the traditional APIT localization algorithm, the RSSI-APIT localization algorithm not only reduced the number of anchor calls and lowered the system’s operating cost, but also effectively improved the localization accuracy and stability of the system, which reduced the localization error by about 57%, and the final error was kept within 1.55 m.

## Data Availability

No new data were created or analyzed in this study. Data sharing is not applicable to this article.
